# Sex and gender differences in drug treatment: experiences from the knowledge database Janusmed Sex and Gender

**DOI:** 10.1186/s13293-023-00511-0

**Published:** 2023-05-12

**Authors:** Linnéa Karlsson Lind, Diana M. Rydberg, Karin Schenck-Gustafsson

**Affiliations:** 1 Health and Medical Care Administration, Region Stockholm, Stockholm, Sweden; 2 Department of Medicine Solna, Karolinska Institutet, Stockholm, Sweden; 3 Clinical Pharmacology Unit, Karolinska University Hospital, Stockholm, Sweden; 4 Centre for Gender Medicine, Department of Medicine Solna, Karolinska Institutet, Stockholm, Sweden; 5 Cardiology Unit, Karolinska University Hospital, Stockholm, Sweden

**Keywords:** Sex differences, Gender differences, Knowledge database, Drug information

## Abstract

**Background:**

Evidence from clinical research indicates that men and women can differ in response to drug treatment. The knowledge database Janusmed Sex and Gender was developed to illuminate potential sex and gender differences in drug therapy and, therefore, achieve a better patient safety. The database contains non-commercial evidence-based information on drug substances regarding sex and gender aspects in patient treatment. Here, we describe our experiences and reflections from collecting, analyzing, and evaluating the evidence.

**Janusmed Sex and Gender:**

Substances have been systematically reviewed and classified in a standardized manner. The classification considers clinically relevant sex and gender differences based on available evidence. Mainly biological sex differences are assessed except for gender differences regarding adverse effects and compliance. Of the 400 substances included in the database, clinically relevant sex differences were found for 20%. Sex-divided data were missing for 22% and no clinically relevant differences were found for more than half of the substances (52%). We noted that pivotal clinical studies often lack sex analyses of efficacy and adverse effects, and post-hoc analyzes are performed instead. Furthermore, most pharmacokinetic analyses use weight correction, but medicines are often prescribed in standard doses. In addition, few studies have sex differences as a primary outcome and some pharmacokinetic analyses are unpublished, which may complicate the classification of evidence.

**Conclusions:**

Our work underlines the need of sex and gender analyses, and sex-divided data in drug treatment, to increase the knowledge about these aspects in drug treatment and contribute to a more individualized patient treatment.

## Highlights

- Sex-analyses of efficacy and safety in studies on drug treatment are still lacking in most cases despite guideline recommendations
- Janusmed Sex and Gender can contribute to a better understanding of the importance in considering patient’s sex, leading to increased patient safety and improved drug treatment
- To improve drug treatment in both men and women, healthcare as well as researchers and regulatory authorities need to address and assess these aspects

## Background

Sex and gender differences have been described for several diseases, especially in cardiovascular medicine [1]. In addition, patient’s sex can influence drug treatment, and men and women may respond differently to the same drug [1]. Historically, women have been excluded from clinical drug trials [2], and therefore, sex-specific information for older drugs is insufficient. Despite recommendations by regulatory authorities to include sex and gender aspects in new drug applications, sex-specific information is often lacking in product information and published data [3]. The need of gathering information on sex and gender differences in drug treatment in a structured manner led to the development of a knowledge database, Janusmed Sex and Gender [4]. In this commentary, we describe our experiences and reflections from collecting, analyzing, and evaluating the evidence.

### Janusmed Sex and Gender

The database provides information about more than 400 drug substances within several therapeutic areas. Mainly sex differences (biological) are presented (pharmacokinetics/dosing/effects/adverse effects). For gender differences (social/cultural), data on drug utilization and adverse effects are discussed when applicable. No data on cultural and socioeconomic factors are included. Data including sexes other than the binary are insufficient. The database is non-commercial and available for Swedish users as well as in English (in total, around 7000 visits/month). The aim of the database is to support physicians and improve drug prescribing with consideration to the patient’s sex. At the initiative of Prof. Karin Schenck-Gustafsson, with funding from The Swedish Association of Local Authorities and Regions (SALAR), the database was developed in 2012–2013 by a joint venture between the Health and Medical Care Administration, Clinical Pharmacology at Karolinska University Hospital and Centre for Gender Medicine at Karolinska Institutet. The development of the database has been described earlier [4]. The database is currently funded by the Health and Medical Care Administration, Region Stockholm, Sweden.

Systematic literature searches are performed with combinations of specific search terms and without a limitation for publication year, as described earlier [4].

The drug substances are classified according to evidence level and clinical relevance; (A) No clinically relevant sex differences, (B) Data on sex differences are lacking or where the data interpretation is complicated, (C) Clinically relevant sex differences in some patient populations, (C!) Clinically relevant sex differences. The classification considers study-type and the quality of the evidence. Historically, some substances used for sex-specific indications (ATC groups G02, G03, G04), were also included in the database, but analyses of sex differences in these cases are not applicable, and, therefore, classified as B.

## Discussion

### Challenges in classifying clinically relevant sex/gender differences

**Fig. 1:**
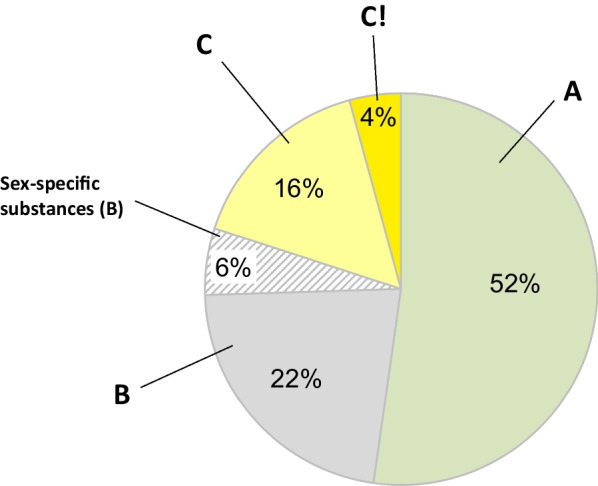
Distribution of drug substances (*n* = 400) according to classification categories; No clinically relevant sex differences (**A**), Data on sex differences are lacking or where the data interpretation is complicated (**B**), Clinically relevant sex differences in some patient populations (**C**), Clinically relevant sex differences (**C!**)

In our database, clinically relevant sex/gender differences were found for 20% of the 400 analyzed substances, mainly regarding efficacy and adverse effects. No clinically important sex/gender differences were found for 52% (Fig. 1). Studies are seldom designed for analyzing sex and gender differences and, therefore, lack statistical power for sex-analyses. Instead, post-hoc analyses are performed. To mitigate this, mandatory inclusion of larger study population (both men and women) in the clinical trials upon registration of new drugs could be part of a solution. A well-represented study population according to the disease prevalence is needed for retrieving relevant sex analyses and for optimization of drug treatment in both men and women. In addition, the study outcome by patient’s sex is often conducted as a subgroup analysis and only available in the supplementary material. An analysis of patient’s sex in a well-done large observational study can have a higher quality of evidence than a clinical study lacking a sex analysis.

In 22% of the analyzed substances, sex and gender data are lacking, manifesting a knowledge gap (Fig. 1). This category consists of mainly older drugs not prioritized in research, and therefore, this knowledge gap will most likely persist. Furthermore, this category also includes drugs, where sex differences in disease symptomatology complicate the assessment. Older drugs were mainly authorized in a regulatory decentralized process, while newer drugs are processed centralized by the EMA [5]. In recent years, sex-divided data in analyses, reporting and publications are required by research councils and academic journals [6]. When searching the literature, we have also observed that reporting of sex-divided data in studies of drug treatment has increased over time. However, the reporting is still insufficient. A recent example is SGLT-2 inhibitors clinical trials, including around 35% women, with a discrepancy to the disease prevalence of HFpEF and lacking sex analyses [7].

Data are for the most part lacking regarding sex hormones used in transgender population, but this is expected to change with time and increased interest in this research field.

### Weight-adjusted dosing and sex differences

Pharmacokinetic studies, normally performed in a small study population, often include sex-divided data although more common for newer drugs. Adjustment for body weight is often used, in contrast to standard dosing used clinically. Although, weight-adjusted doses might explain some differences between men and women. Even after weight adjustment, sex differences in pharmacokinetics have not been considered clinically relevant in most cases. For drugs mainly renally excreted (e.g., digoxin, pregabalin and ganciclovir), pharmacokinetic sex differences might be more clinically important than previously believed [8].

### Patient’s sex in relation to adverse events

Prevalence of adverse events are more common in women [9, 10], partially explained by generally lower body weight and lower renal excretion in women which could lead to higher dose exposure [8, 11]. Other explanatory factors could be more prevalent drug utilization [12], polypharmacy and drug–drug interactions [13], and higher prevalence of adverse event reporting [14] in women. The most common type of adverse events are the predictable and preventable dose-dependents adverse events (type A). The more rare and unpredictable adverse events (type B) are more common in women, probably due to higher immunoreactivity and influence of sex hormones [15].

The increased risk of Torsade de Pointes in women induced by drugs prolonging the QT-interval, is a well-described adverse event, caused by women having a longer QT-interval in general [16]. This has led to withdrawal of drugs by regulatory authorities [17]. Testosterone can shorten the QT-interval and may, therefore, reduce the risk of this adverse event in men [16]. Example of drugs with the potential of inducing this type of ventricular tachycardia are, amiodarone, sotalol, and erythromycin [16].

### Reproductive factors can affect drug treatment

Physiological changes during pregnancy can affect the plasma levels of drugs, such as lamotrigin and topiramate, requiring frequent monitoring and dose adjustment [18]. On the other hand, drugs such as carbamazepine and phenytoin can have a negative influence on endogenous sex hormones in both men and women, impacting sexual health and menstruation [18]. Furthermore, cancer treatment such as capecitabine and fluorouracil can induce changes in the sex cells for both men and women, and therefore, effective contraceptives are necessary during and following cancer treatment [19]. Synthetic estrogens and progestogens can influence plasma levels of certain drugs, such as carbamazepine and ritonavir [20].

## Conclusions

Janusmed Sex and Gender is a unique knowledge database with information on sex and gender aspects in drug treatment. Despite the requirements of including both sexes in research, and an increased reporting of sex-divided results, these data are still inadequate in published studies, although it has improved over time. For some drugs, there is evidence of sex and gender differences in efficacy or adverse effects; however, patient’s sex is rarely considered in treatment guidelines or drug product information. Our database highlights the importance of considering sex and gender analyses in both drug development, treatment, and clinical use. Hopefully this will lead to a better understanding of sex and gender influence on drug treatment and improved individualized treatment of both men and women.

## Data Availability

Janusmed Sex and Gender is available at https://www.janusinfo.se/genus/in-english.
